# An Al_2_O_3_ Gating Substrate for the Greater Performance of Field Effect Transistors Based on Two-Dimensional Materials

**DOI:** 10.3390/nano7100286

**Published:** 2017-09-22

**Authors:** Hang Yang, Shiqiao Qin, Xiaoming Zheng, Guang Wang, Yuan Tan, Gang Peng, Xueao Zhang

**Affiliations:** 1 College of Science, National University of Defense Technology, Changsha 410073, China; yanghangnudt@163.com (H.Y.); 15874954147@163.com (X.Z.); wangguang@nudt.edu.cn (G.W.); tanyuany123@126.com (Y.T.); 2 College of Optoelectronic Science and Engineering, National University of Defense Technology, Changsha 410073, China; sqqin8@nudt.edu.cn; 3 College of Physics and Electronics, Central South University, Changsha 410073, China

**Keywords:** graphene, WS_2_, Al_2_O_3_ gating substrate, field effect transistors

## Abstract

We fabricated 70 nm Al_2_O_3_ gated field effect transistors based on two-dimensional (2D) materials and characterized their optical and electrical properties. Studies show that the optical contrast of monolayer graphene on an Al_2_O_3_/Si substrate is superior to that on a traditional 300 nm SiO_2_/Si substrate (2.4 times). Significantly, the transconductance of monolayer graphene transistors on the Al_2_O_3_/Si substrate shows an approximately 10-fold increase, due to a smaller dielectric thickness and a higher dielectric constant. Furthermore, this substrate is also suitable for other 2D materials, such as WS_2_, and can enhance the transconductance remarkably by 61.3 times. These results demonstrate a new and ideal substrate for the fabrication of 2D materials-based electronic logic devices.

## 1. Introduction

Two-dimensional (2D) materials, such as graphene and transition-metal dischalcogenides (TMDs), have attracted tremendous interest for possible applications in transistors [1,2,3,4], photodetectors [5,6], and touch panels [7,8] owing to their extraordinary properties. However, most efforts to date employ a 300 nm thick silicon dioxide (SiO_2_) substrate as the gate dielectric layer. This substrate is widely used is mainly because 2D materials can be readily visualized using an optical microscope due to optical interference [9,10,11]. Although they have led to many interesting scientific discoveries [12,13,14], applying 300 nm SiO_2_ substrates will greatly reduce the performance of the devices, especially the signal amplification capability, which is one of the most important parameters of contemporary integrated circuits [15,16]. The devices fabricated on SiO_2_/Si substrates lack enough capability to regulate the Fermi surface of samples, thus requiring higher back-gate voltage [15]. Generally, the top-gate structure is adopted to enhance the gate capacitance of field effect transistors (FETs). However, its fabrication is challenging, as 2D materials lack dangling bonds [17,18]. Although many alternate approaches have been demonstrated, they inevitably result in the degradation of carrier mobility [19,20].

Previously, Liao et al. firstly reported that applying 72 nm Al_2_O_3_/Si substrates could improve the optical contrast and electrical properties of single-graphene FETs [21]. However, the Al_2_O_3_ film they fabricated was not well insulated since they directly attached tape onto the Al_2_O_3_/Si substrate using mechanical exfoliation [6]. This conventional method may damage Al_2_O_3_ films due to the strong adhesive force of the tape [21]. In our experiments, the deterministic transfer method was applied to transfer graphene onto an Al_2_O_3_/Si substrate to avoid this problem. In addition, the corrosion time of the HF solution was carefully controlled so that the wafer would be smoother, which was beneficial for forming a dense Al_2_O_3_ film via atomic layer deposition (ALD) growth. Accordingly, our single-layer graphene FETs, compared to those of Liao’s work, achieved a greater performance. Furthermore, we systematically investigated WS_2_ FETs on an Al_2_O_3_ gating substrate, showing that this superior substrate is also suitable for other 2D materials. 

## 2. Experimental Details

**Figure 1:**
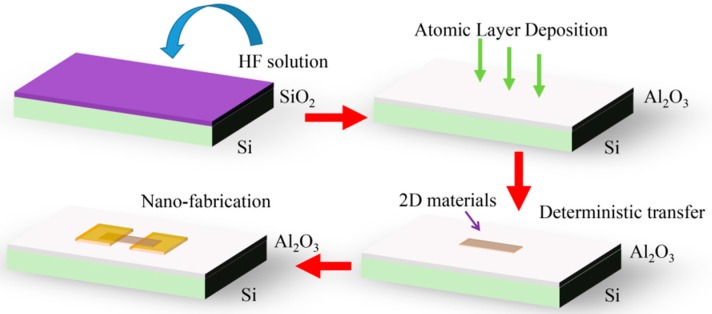
The process of fabricating graphene (or WS_2_) field effect transistors (FETs) on Al_2_O_3_/Si substrates. HF: hydrofluoric acid.

Figure 1 illustrates the fabrication process of graphene- (or WS_2_)-based FETs on Al_2_O_3_/Si substrates. Firstly, an Al_2_O_3_ film was deposited on silicon (doped n++, conductivity: 0.01–0.02 Ω·cm) wafers via the ALD technique using tri-methyl-aluminum ((CH_3_)_3_Al) and distilled water as the source (reaction temperature: 250 °C). Prior to the deposition of Al_2_O_3_, a native SiO_2_ layer was removed with a 5% (mole ratio) hydrofluoric acid (HF) solution (40 min). After the Al_2_O_3_ film was deposited, graphene (or WS_2_) was transferred onto that substrate via the deterministic transfer method [22] (see Figure S1). At last, the source and drain contacts were patterned using e-beam lithography (EHT: 10 kV, aperture size: 30 μm, beam current: 217.1 pA), and 10 nm Ti/50 nm Au were deposited using e-beam evaporation (vacuum: 1 × 10^−5^ Pa, evaporation rate: Ti: 0.5 Å/s; Au: 1.5 Å/s).

The topography of the samples was characterized via atomic force microscopy (AFM, NT-MDT company, Moscow, Russia, scanning mode: Semi-contact, scanning frequency: 1.01 (Hz), scanning electron microscopy (SEM, Raith company, Dortmund, Germany) and optical microscopy (Nikon company, Tokyo, Japan). The Raman and contrast spectra were recorded with Confocal Raman Spectrometer (WiTec company, Ulm, Germany, exciting laser wavelength: 532 nm, spot size: 2 μm). The thickness of the Al_2_O_3_ film was obtained with GES-5 ellipsometer (Sopra Company, Annecy, France) and calculated to be approximately 70 nm. All characterizations were conducted in ambient conditions and at room temperature (300 K). The electrical properties were measured with 4200-SCS probe system (Keithley Company, Cleveland, OH, USA).

## 3. Results and Discussion

**Figure 2:**
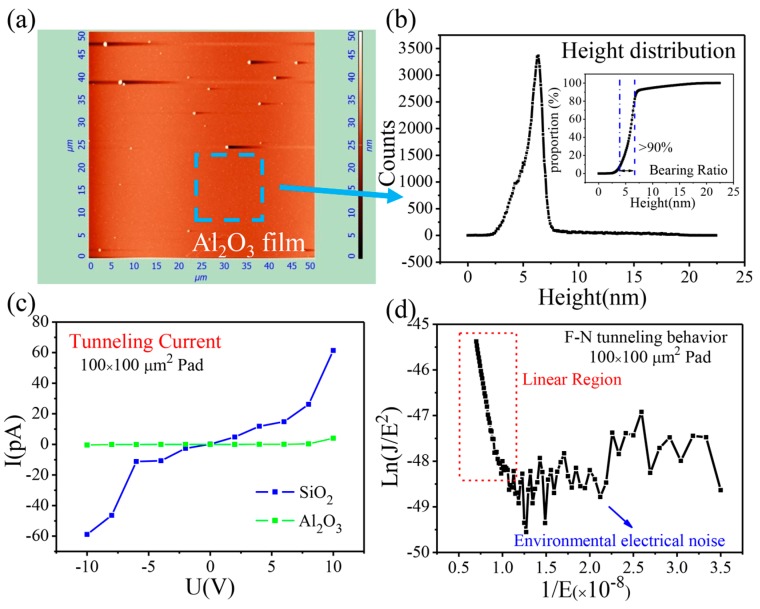
Characterization of 70 nm Al_2_O_3_ film prepared by atomic layer deposition (ALD). (**a**) Atomic force microscopy (AFM) image and corresponding (**b**) height distribution of film surface (areas in blue dashed box). (**c**) Tunneling currents of Al_2_O_3_ and SiO_2_ films. (**d**) Flow–Nordheim (F–N) fitting curve of the metal-insulator-semiconductor (MIS) device.

As shown in Figure 2a, the Al_2_O_3_ film is uniform over a large area (50 μm × 50 μm). Figure 2b illustrates the height distribution of the local area, which mainly varies from 4 to 6 nm. The parameters of surface roughness are given in Table S1. Based on the measurements, the Si surface is extremely smooth after HF treatment. In addition, the average surface roughness of the ALD-grown Al_2_O_3_ film is 1.26 nm.

To understand the dielectric properties of the Al_2_O_3_ film, I-V characteristics were firstly measured based on metal-insulator-semiconductor (MIS) devices with Al_2_O_3_ and SiO_2_ insulating layers on silicon wafers (shown in Figure 2c). When bias voltage increased to 10 V, the tunneling current of Al_2_O_3_ was only one tenth of that of SiO_2_. This indicates that the Al_2_O_3_ dielectric layer can withstand a higher gate voltage, resulting in greater modulation of the Fermi level of 2D materials. In general, the I-V characteristic of the dielectric layer can be described via Fowler–Nordheim (F–N) tunneling behavior [21,23]:

(1) $$J=AE_{ox}^{2}\exp(-B/E_{ox})$$

where *J* is current density, *E_ox_* is the electric field, and *A* and *B* are constants considering carrier effective mass and barrier height, respectively. Apparently, based on Figure 2d, when the electric field is large, it is in good agreement with the theoretical model [24]. However, in the case of small electric fields, due to the influence of electrical noise in the environment, the experimental curve exhibits fluctuation [19].

**Figure 3:**
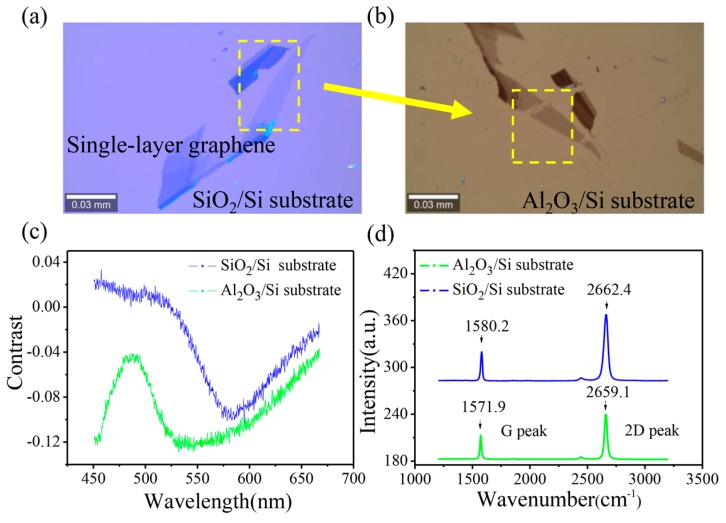
(**a**,**b**) Optical image of graphene on the SiO_2_/Si and Al_2_O_3_/Si substrates. (**c**) The contrast and (**d**) Raman spectra of graphene on SiO_2_/Si and Al_2_O_3_/Si substrates. Raw data and processing methods are shown in Figures S2 and S3.

Optical contrast is the difference in visual properties that enables us to distinguish an object from other objects and the background. Figure 3a,b shows the optical image of graphene on SiO_2_/Si and Al_2_O_3_/Si substrates, respectively. To quantify the contrast of graphene on different substrates, the color images are converted to gray-scale images. By calculation [25], the absolute value of contrast intensity of graphene on the Al_2_O_3_/Si substrate (−0.12) is significantly higher than that on the SiO_2_/Si substrate (−0.05). Furthermore, from the contrast spectrum shown in Figure 3c, the absolute value of the contrast on the Al_2_O_3_/Si substrate in the 450~700 nm wavelength range is always higher than that on the SiO_2_/Si substrate. The best contrast of graphene on the Al_2_O_3_/Si substrate could be obtained with 450 nm and 550 nm illuminations. As depicted in Figure 3d, the G peak and the 2D peak of graphene on the Al_2_O_3_/Si substrate experience a red-shift (8.3 cm^−1^ for G peak and 3.3 cm^−1^ for 2D peak). The Raman shift could be simplified with the harmonic oscillator model [26]: $\Delta k=\sqrt{\beta /m}$, where Δk is the Raman shift, β is the mechanical constant, and *m* is the effective mass. Because of the presence of spotted islands on the Al_2_O_3_/Si substrate, a tensile stress is formed onto graphene, which leads to a decrease in β and subsequently the red-shift of the Raman vibration peak of graphene on the Al_2_O_3_/Si substrate [26].

**Figure 4:**
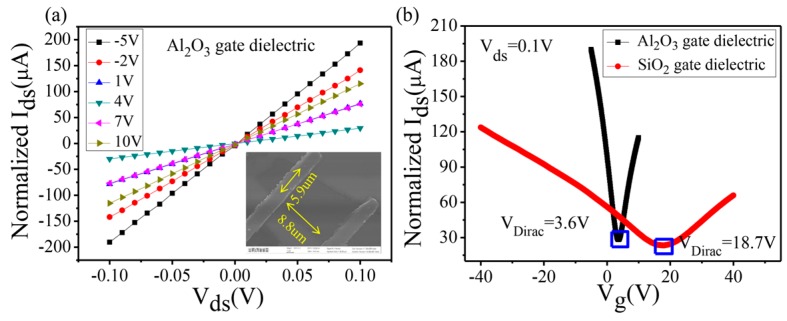
(**a**) Output characteristics of graphene FETs on the Al_2_O_3_/Si substrate at different gate voltages (−5~10 V). The inset shows an SEM image of the device. (**b**) Transfer characteristics of graphene FETs on different substrates.

Next, the electrical properties of graphene FETs on the Al_2_O_3_/Si substrate were studied in nitrogen. As depicted in Figure 4a, the drain-source current (*I_ds_*) increases linearly in pace with the bias voltage, indicating good ohmic contact between the graphene and the electrode. The aspect ratio (*L*/*W*) of the channel is approximately 1.5, as shown in the SEM image. For better comparison among different samples, normalized *I_ds_* (=*I_ds_* ×L/W) was applied, which considered the influence of the aspect ratio. Figure 4b shows the transfer characteristics of our devices (*V_g_* means back-gate voltage). It is obvious that the curve slope of the device on the Al_2_O_3_/Si substrate is significantly higher than that on the SiO_2_/Si substrate, indicating a greater gate regulation ability of the 70 nm Al_2_O_3_ dielectric layer. In addition, for graphene on the Al_2_O_3_/Si substrate, when the gate voltage increases from −5 to 3.6 V, the current decreases from 190 to 28.3 A, so the unit on/off ratio is evaluated to be 0.78 V^−1^. However, the unit on/off ratio for graphene on the SiO_2_/Si substrate reaches only 0.09 V^−1^. Accordingly, the magnification capability was easily estimated to increase by 8.7 times. The minimum conductance on the Al_2_O_3_/Si substrate is slightly higher than that on the SiO_2_/Si substrate, which may be due to induced impurities in the transfer process, leading to more carriers in grapheme [8]. For further discussion, some significant parameters of FETs are listed in Table 1.

The normalized transconductance *g_m_* can be extracted from the following [27]:

(2) $$g_{m}=\frac{dI_{ds}}{dV_{g}}\frac{L}{W}$$

**Figure 5:**
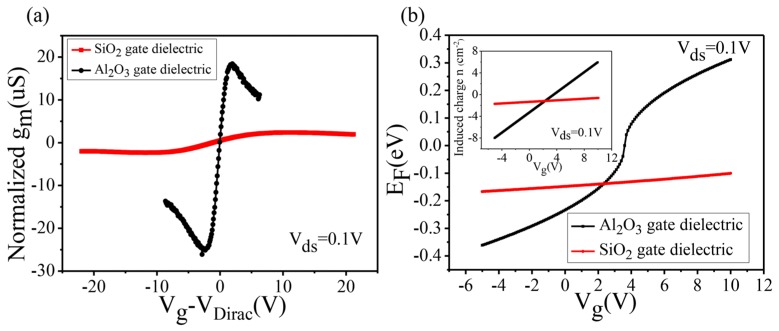
(**a**) *g_m_*–*V_g_* curves of graphene FETs on different substrates. (**b**) *E_F_*–*V_g_* curves of graphene FETs on different substrates. The inset shows the variation tendency of the induced charge against gate voltage.

The black curve in Figure 5a illustrates the transconductance variation of graphene on the Al_2_O_3_/Si substrate. It can be seen that the maximum negative transconductance and maximum positive transconductance are −26.1 μS (*V_g_* = −3.1 V) and 19.4 μS (*V_g_* = 2.9 V), respectively. Compared with the maximum *g_m_* of graphene on the SiO_2_/Si substrate (2.6 μS), it can be concluded that the regulation ability of the Al_2_O_3_ dielectric layer is about 10 times that of SiO_2_, which is in accordance with previous estimations. Accordingly, the value of effective dielectric constant for Al_2_O_3_ is 9.2, which is consistent with the theoretical dielectric constant (8~10) of the Al_2_O_3_ film grown by ALD [28]. The changes of the Fermi level of graphene can be fitted with the theoretical model [29]:

(3) $$E_{F}=hv_{F}\sqrt{\pi n}/2\pi q=hv_{F}\sqrt{\pi \varepsilon_{0}\varepsilon (V_{g}-V_{D})/qd}/2\pi q$$

where *E_F_* is the Fermi level, *n* is the induced charge amount, *h* is the Planck constant, $v_{F}$ is the Fermi speed, *q* is the elementary charge, and *V_D_* is the Dirac point. The amount of charges induced by the applied gate voltages on different substrates is shown in the inset of Figure 5b. Obviously, as the thickness decreases and the dielectric constant increases, the shift of the Fermi level of graphene on the Al_2_O_3_/Si substrate is far greater than that on the SiO_2_/Si substrate. In order to evaluate the mobility of the devices, a device model was used [27]. The extracted carrier mobility of graphene FETs on the Al_2_O_3_/Si substrate is 6500 cm^2^ V^−1^·s^−1^, which is similar to 6780 cm^2^ V^−1^·s^−1^ of the FETs on the SiO_2_/Si substrate. The replacement of the substrate does not lead to the degradation of the transport performance of the devices. Furthermore, transfer characteristic of few-layer graphene FETs on an Al_2_O_3_/Si substrate was depicted in Figure S4, showing that Al_2_O_3_ gating substrate is also suitable for few-layer graphene.

**Figure 6:**
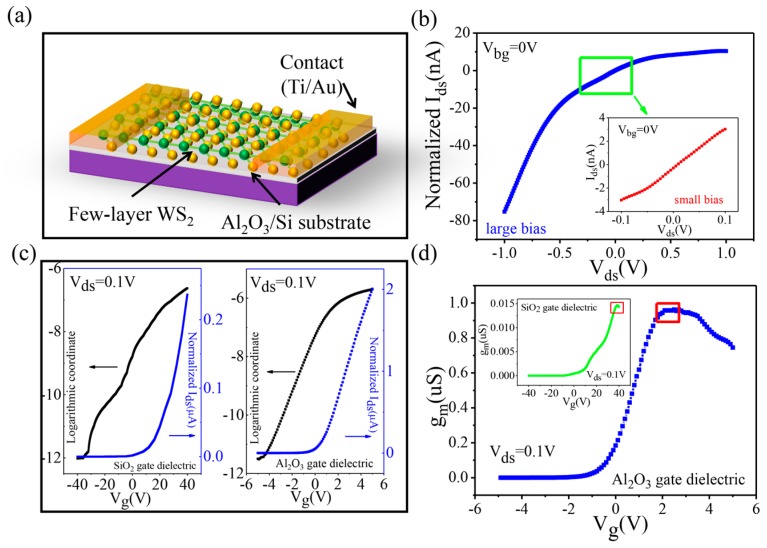
(**a**) Three-dimensional schematic view of the few-layer WS_2_ FETs. (**b**) Output characteristics of few-layer WS_2_ on Al_2_O_3_/Si substrates in large bias and (inset) small bias voltage. (**c**) Transfer characteristics of few-layer WS_2_ FETs on different substrates. (**d**) *g*_m_–*V*_g_ curves of few-layer WS_2_ FETs on different substrates.

We also systematically studied the electrical properties of few-layer WS_2_ on the Al_2_O_3_/Si substrate (Figure 6a). The number of layers was determined by Raman and Photoluminescence spectra (see Figure S5). A single layer was not used because the surface states of single-layer TMDs are easily affected by the external environment in the process of device fabrication, thus losing the intrinsic property [30]. As Figure 6b shows, the current varies nonlinearly against the change in bias voltage from −1 to 1 V. This is due to the formation of the Schottky barrier between WS_2_ and metal contact, which was widely reported in previous studies [30,31,32]. However, from the inset of Figure 6b, it can be seen that the linearity is maintained fairly well under the condition of small bias voltage. Hence, the bias voltage is maintained at 0.1 V in the following test. 

Figure 6c illustrates the transfer characteristics of few-layer WS_2_ on different substrates, and both of them distinctly exhibit n-type behavior conduction [31]. When the gate voltage changes from −10 to 10 V, devices on Al_2_O_3_/Si substrates turn from the off state (2.8 pA) to the on state (2.5 μA). Therefore, the unit on/off ratio is as highly as 10^5^ V^−1^, which is far greater than that on the SiO_2_/Si substrate (1.5 × 10^3^ V^−1^). As depicted in Figure 6d, the maximum transconductance (red rectangle) of few-layer WS_2_ can reach about 0.92 μS (V_g_ = 2.3 V), and the corresponding carrier mobility is calculated to be 239 cm^2^·V^−1^·s^−1^. However, as the gate voltage continues to increase, the transconductance starts to decline, indicating that the carrier mobility has reached the maximum value. Compared with the maximum transconductance of few-layer WS_2_ on the Si/SiO_2_ substrate (1.5 × 10^−2^ μS), the gate control ability was significantly improved (61.3 times). Furthermore, the Al_2_O_3_ substrate is a better alternative for other 2D materials (such as WS_2_). 

## 4. Conclusions

In summary, Al_2_O_3_/Si substrates are superior for the visualization of graphene and fabrication of graphene transistors. Compared with SiO_2_/Si substrates, Al_2_O_3_/Si substrates can enhance the optical contrast of graphene by up to 2.4 times. Furthermore, using the Al_2_O_3_ film as the gate dielectric, the transconductance of graphene FETs exhibited an approximately 10-fold increase. Significantly, this substrate is also more suitable for other 2D materials, such as WS_2_, and can remarkably enhance the transconductance by 61.3 times. 

## Figures and Tables

**Table 1:** Significant parameters of graphene FETs on different substrates.

	Parameters	Minimum Conductivity	Dirac Point	Maximum Trascondutance	Mobility
Substrate					
Al_2_O_3_		283 μS	3.6 V	−26.1 μS	6500 cm^2^ V^−1^·s^−1^
SiO_2_		237 μS	18.7 V	2.6 μS	6780 cm^2^ V^−1^·s^−1^

## Supplementary Materials

The following are available online at http://www.mdpi.com/2079-4991/7/10/286/s1, Figure S1: Steps of deterministic transfer method. Figure S2: Reflection spectra of graphene on different substrates. Figure S3: Raman scanning image of graphene on different substrates. Figure S4: Transfer characteristic and Raman spectrum of few-layer graphene on an Al_2_O_3_/Si substrate. Figure S5: Photoluminescence and Raman spectra of few-layer WS_2_ on Al_2_O_3_/Si substrate. Table S1: Average roughness (Sa), root mean square (Sq) and coefficient of kurtosis (Ska) of three different substrates (Si, SiO_2_, Al_2_O_3_).
