# Supplementary material for: An Al2O3 Gating Substrate for the Greater Performance of Field Effect Transistors Based on Two-Dimensional Materials
Source: Nanomaterials (Basel). 2017 Sep 22;7(10):286. doi: 10.3390/nano7100286 (PMC5666451; doi:10.3390/nano7100286)
Supplement: Supplementary file 1 [file nanomaterials-07-00286-s001.pdf]

Electronic Supplementary Information

An Al_2O_3 Gating Substrate for the Greater Performance of Field Effect Transistors Based on Two-Dimensional Materials

Hang Yang[1], Shiqiao Qin[2], Xiaoming Zheng[1,3], Guang Wang[1], Yuan Tan[1], Gang Peng[1*], and Xueao Zhang[1*]

[1]*College of Science, National University of Defense Technology, China*

[2]*College of Optoelectronic Science and Engineering, National University of Defense Technology, China*

[3]*College of Physics and Electronics, Central South University, China*

[*].Corresponding author. *E-mail address:* penggang@nudt.edu.cn

[*].Corresponding author. *E-mail address:* xazhang@nudt.edu.cn

Experimental details

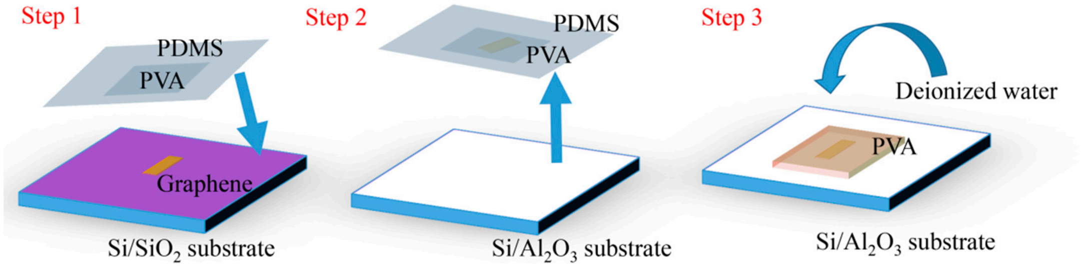

Figure S1. Steps of deterministic transfer method. Graphene (or WS_2) nanoflakes were firstly exfoliated from the graphite crystals onto SiO_2/Si substrates using the mechanical exfoliation technique[1]. Then polymer layers (polydimethylsiloxane (PDMS) and polyvinyl alcohol (PVA)) are used to transfer graphene nanoflakes from Si/SiO_2 substrate to PVA by strong adhere force between samples and polymer layer (heat temperature: 80℃). The polymer layers are then mounted in a micromanipulator to transfer the graphene flakes to Al_2O_3/Si substrate. After transfer, the sacrificial polymer layer (PVA) is dissolved with deionized water[2].

Results and Discussion

parameters \ substrate	Si	SiO_2	Al_2O_3
**Sa**	0.18	0.30	1.26
**Sq**	0.27	0.60	2.24
**Ska**	301.4	45.3	11.3

Table S1. Average roughness (Sa), root mean square (Sq) and coefficient of kurtosis (Ska) of three different substrates (Si, SiO_2, Al_2O_3). Obviously, the silica surface after washing by HF seems extremely smooth, just like original silicon. While some spotted islands distribute on Al_2O_3 surface, overall it is relatively flat (Sa=1.26nm).

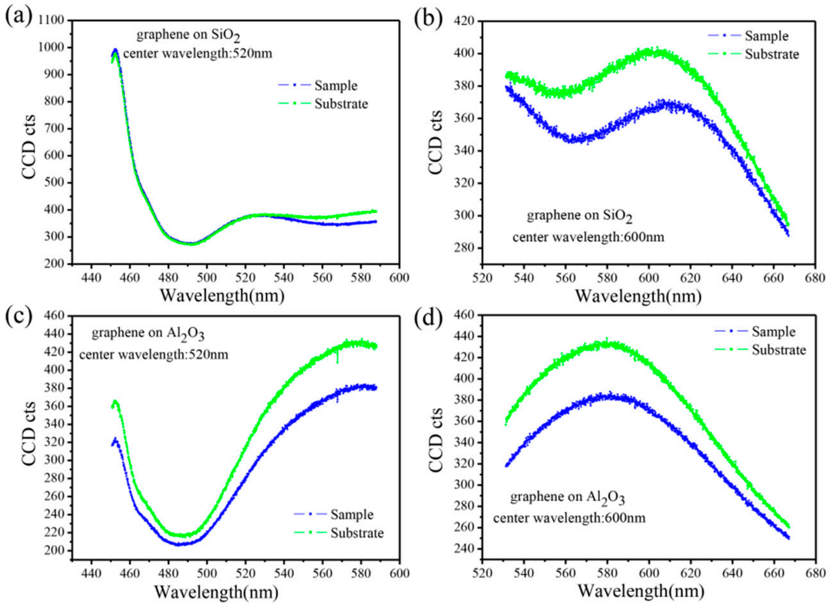

Figure S2. Reflection spectra of graphene on different substrates. Here, the brightness of the light source was kept to the same all the time. Due to the limitation of grating range, tests are performed at two center wavelengths: 520 nm and 600 nm. Finally, contrast of graphene on different substrates was extracted by[3]: Contrast=(R_{sample}-$R_{substrate}$)/R_{sample}.. The contrast spectrum is smoothed by Savitzky-Golay method (parameters: left:

5, right: 5, order: 4).

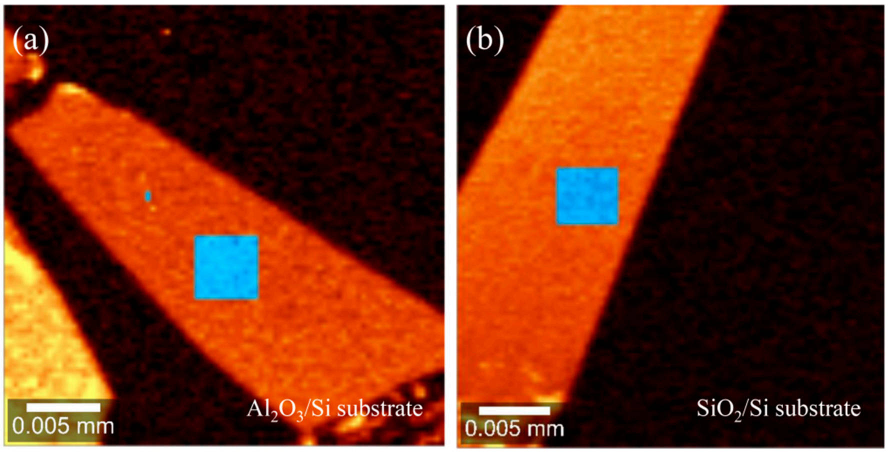

Figure S3. Raman scanning image of graphene on different substrates. In practical situation, Raman spectrum of each point on graphene is not exactly the same. It would fluctuate due to the adsorbent of water vaper or oxygen molecules on graphene surface. Accordingly, a method named average selective area was adopted. Raman imaging of graphene (center: 2700 cm^{-1}, width: 300 cm^{-1}) was conducted at first. And then a homogeneous region was selected (blue box shown in figure) to analysis. Finally, average Raman spectrums of graphene on different substrates were obtained.

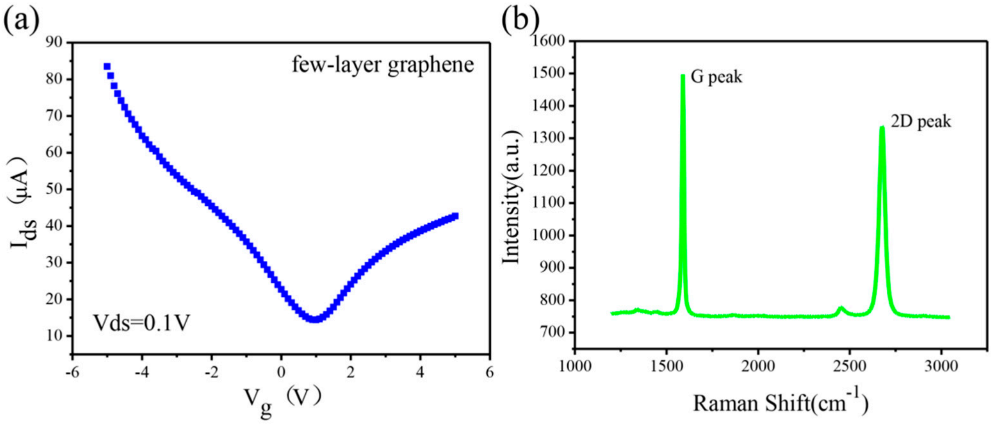

Figure S4. (a) Transfer characteristic and (b) Raman spectrum of few-layer graphene on an Al_2O_3/Si substrate. Obviously, Al_2O_3 dielectric layer owns capability to regulate Fermi level of few-layer graphene. As shown in figure, when the gate voltage changes from -5 V to 1 V, the on/off ratio can reach about 9. Therefore the unit on/off ratio is 1.5 V^{-1}, which is higher than that of monolayer graphene (0.78 V^{-1}). Generally speaking, few-layer graphene has a band gap between the conduction band and the valence band[4], thereby the unit on/off ratio is relatively higher than monolayer graphene, but the carrier mobility will be accordingly lower.

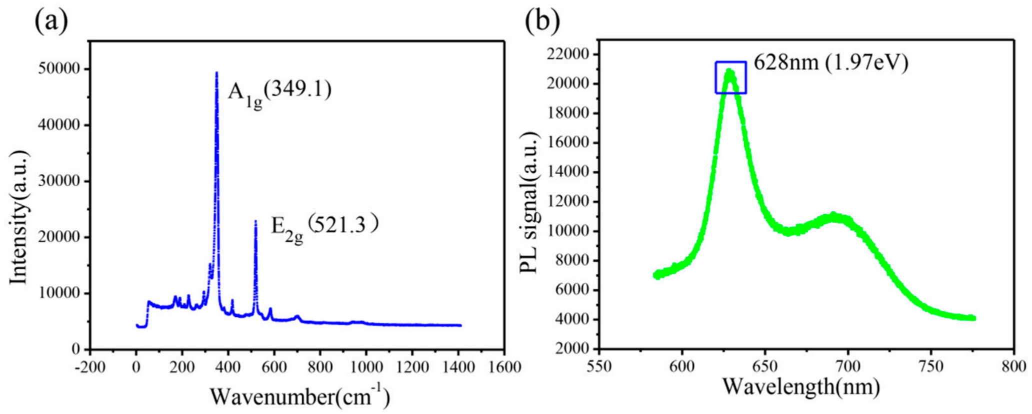

Figure S5. (a) Photoluminescence and (b) Raman spectra of few-layer WS_2 on Al_2O_3/Si substrate. WS_2 is an indirect-bandgap semiconductor in bulk state, but can turn into a direct-bandgap material when exfoliated into the monolayer state. As shown in (a), the PL intensity of few-layer WS_2 reach maximum at 1.97eV. Besides, exfoliated few-layer WS_2 flakes have shown A_{1g} (in-plane optical mode) and E_{2g} (out-of-plane vibrations of the molybdenum atoms) located at 349.1 cm^{-1} and 521.3 cm^{-1}, which is consistent with earlier studies[5].

References

[1] Novoselov K S, Geim A K, Morozov S V, et al. Electric field effect in atomically thin carbon films [J]. science, 2004, (306): 666-9.

[2] Dean C R, Young A F, Meric I, et al. Boron nitride substrates for high-quality graphene electronics [J]. Nature Nanotechnology, 2010, (5): 722.

[3] Ni Z, Wang H, Kasim J, et al. Graphene thickness determination using reflection and contrast spectroscopy [J]. Nano letters, 2007, (7): 2758-63.

[4] Hong X, Posadas A, Zou K, et al. High-mobility few-layer graphene field effect transistors fabricated on epitaxial ferroelectric gate oxides [J]. Physical review letters, 2009, (102): 136808.

[5] Berkdemir A, Gutiérrez H R, Botelloméndez A R, et al. Identification of individual and few layers of WS_2 using Raman Spectroscopy [J]. Scientific Reports, 2013, (3): 1755.
